# Genetische, endokrine, medikamentöse und pankreoprive Diabetesformen und exokrine Pankreasinsuffizienz (Update 2026)

**DOI:** 10.1007/s00508-025-02653-0

**Published:** 2026-04-30

**Authors:** Susanne Kaser, Sabine E. Hofer, Lili Kazemi-Shirazi, Thomas Stulnig, Yvonne Winhofer-Stöckl, Michael Resl, Claudia Ress, Harald Sourij, Felix Aberer, Gerlies Treiber, Adrian Szodl, Elisabeth Binder, Bernhard Radlinger, Anton Luger

**Affiliations:** 1 Universitätsklinik für Innere Medizin I Innsbruck, Medizinische Universität Innsbruck, Innsbruck, Österreich; 2 Universitätsklinik für Pädiatrie 1, Medizinische Universität Innsbruck, Innsbruck, Österreich; 3 Klinische Abteilung für Gastroenterologie und Hepatologie, Universitätsklinik für Innere Medizin III, Medizinische Universität Wien, Wien, Österreich; 4 3. Medizinische Abteilung und Karl Landsteiner Institut für Stoffwechselerkrankungen und Nephrologie, Klinik Hietzing, Wien, Österreich; 5 Klinische Abteilung für Endokrinologie und Stoffwechsel, Universitätsklinik für Innere Medizin III, Medizinische Universität Wien, Wien, Österreich; 6 Abteilung für Innere Medizin, Konventhospital der Barmherzigen Brüder Linz, Linz, Österreich; 7 Klinische Abteilung für Endokrinologie und Diabetologie, Universitätsklinik für Innere Medizin, Medizinische Universität Graz, Graz, Österreich; 8 Abteilung für Innere Medizin II, Barmherzige Brüder Krankenhaus Eisenstadt, Eisenstadt, Österreich

**Keywords:** Spezifische Diabetesformen, Exokrine Pankreasinsuffizienz, Genetische Diabetesformen, Medikamentös induzierte Diabetesformen, Diabetes im Rahmen anderer endokrinologischer Erkrankungen, Specific diabetes types, Exocrine pancreatic insufficiency, Genetic diabetes forms, Drug-induced diabetes, Diabetes due to other endocrine disorders

## Abstract

Die in der Diabetes-Klassifikation als Typ-3-Diabetes beschriebenen Krankheitsentitäten umfassen andere spezifische Diabetesformen, welche pathophysiologisch und therapeutisch eine sehr heterogene Krankheitsgruppe darstellen. Diese beinhalten Formen, die aufgrund von genetischen Erkrankungen (monogenetische Formen, neonataler Diabetes, Down-Syndrom, Klinefelter-Syndrom, Turner-Syndrom) oder im Rahmen anderer endokrinologischer Erkrankungen auftreten (z. B. Akromegalie, Cushing-Syndrom, Glukagonom), sowie die an Häufigkeit und Komplexität zunehmenden medikamentös induzierten Diabetesformen (z. B. Therapie mit Glukokortikoiden, Immun-Checkpoint-Inhibitoren, Calcineurininhibitoren, Phosphatidylinositol-3-Kinase-Inhibitoren, hochaktive antiretrovirale Therapie [HAART], Antipsychotika). Ebenfalls in diese Diabeteskategorie fallen pankreoprive Formen (z. B. postoperativ, Zustand nach Pankreatitis, Pankreastumoren, Hämochromatose, zystische Fibrose), seltener infektionsgetriggerte (z. B. kongenitale Rötelninfektion) oder autoimmune Formen (z. B. Stiffman-Syndrom, Anti-Insulin-Rezeptor-Antikörper, Insulin-Autoimmunsyndrom). Die korrekte Zuordnung des Diabetestyps hat unmittelbare therapeutische Auswirkungen für die Betroffenen. Zusätzlich findet sich nicht nur bei pankreopriven Formen, sondern auch bei Diabetes mellitus Typ 1 oder langjährigem Typ-2-Diabetes mellitus häufig eine Assoziation mit einer exokrinen Pankreasinsuffizienz.

## Abstract (en)

Diabetes classified as category 3, usually referred to as type 3 diabetes, encompasses specific types due to other causes. These specific forms of diabetes are clinically and pathophysiologically very heterogeneous. Type 3 diabetes encompasses inherited forms of diabetes (monogenetic diabetes, neonatal diabetes, Down syndrome, Klinefelter syndrome, Turner syndrome), diabetes due to other endocrine disorders (acromegaly, Cushingʼs disease, glucagonoma) and drug-induced forms, e.g., caused by glucocorticoids, immune checkpoint inhibitors, calcineurin inhibitors, phosphatidylinositol 3 kinase inhibitors, highly active antiretroviral therapy (HAART) and antipsychotics. Additionally, this category includes pancreatogenic forms (after pancreatic surgery, after pancreatitis, pancreatic tumors, hemochromatosis, cystic fibrosis), rare infection-triggered (e.g., congenital rubella syndrome) and autoimmune forms other than type 1 diabetes (e.g., Stiffman syndrome, anti-insulin receptor antibodies, insulin autoimmune syndrome). An exact diagnosis is critical for correct and optimal treatment of affected patients. Exocrine pancreatic insufficiency is not only found in patients with pancreatogenic diabetes but also in patients with type 1 diabetes or long-standing type 2 diabetes.

## I) Genetische Diabetesformen

Unter genetischen Formen wird ein heterogenes Krankheitsbild zusammengefasst.

Am häufigsten handelt es sich um erblich bedingte Diabetesformen zurückzuführen auf eine β‑Zell-Dysfunktion (MODY) oder aber eine Glukosestoffwechselstörung im Rahmen von anderen genetischen Erkrankungen (s. bitte auch Absatz Insulinresistenz und Diabetes bei Lipodystrophiesyndromen). Selten sind genetische Störungen der Insulinwirkung (Tab. 1).

**Tab. 1:** Übersicht über genetische Diabetesformen. (Adaptiert von [86]) [138]

Genetische Störungen der Betazellfunktion	MODY
	Transienter oder permanenter neonataler Diabetes
	Mitochondriale Diabetesformen
	Andere
Genetische Störungen der Insulinwirkung	Typ-A-Insulinresistenz
	Leprechaunismus
	Rabson-Mendenhall-Syndrom
	Lipoatropher Diabetes
	Andere
Mit Diabetes mellitus assoziierte andere genetische Erkrankungen	Down-Syndrom
	Klinefelter-Syndrom
	Turner-Syndrom
	Wolfram-Syndrom
	Friedreich-Ataxie
	Chorea Huntington
	Laurence-Moon-Biedl-Syndrom
	Dystrophia myotonica
	Porphyrie
	Prader-Willi-Syndrom
	Andere

Die durchaus nicht selbsterklärende Bezeichnung „maturity-onset diabetes of the young“ (MODY) erfordert den historischen Kontext; sie beruht auf der in den 1960er-Jahren strikt geltenden Annahme zweier Diabetesformen, einer im jungen Alter auftretenden Form, die einer Insulintherapie bedarf (heute: Typ-1-Diabetes) und einer „milden“ Diabetesform, die typischerweise im Erwachsenenalter auftritt (heute: Typ-2-Diabetes) [1]. Im Jahr 1964 wurde erstmals der Begriff „maturity-onset type diabetes of childhood or of the young“ als Beschreibung einer familiären, milden Diabetesform geprägt und 10 Jahre später der autosomal-dominante Erbgang dieser Diabetesform, die auch bereits im Kindes- und jungen Erwachsenenalter zu diagnostizieren war, bestätigt [2, 3].

Nach heutigem Wissen erscheint die Bezeichnung „monogenetischer“ Diabetes (gegenüber der historischen Bezeichnung „maturity-onset diabetes of the young“) sinnvoller. Weiters rückt die aktuelle Literatur von der ursprünglichen numerischen Bezeichnung der MODY-Subtypen ab; so wird etwa die vormals als MODY 3 bezeichnete Form heute – der zugrunde liegenden Mutation entsprechend – als HNF1A-MODY bezeichnet [4]. Aufgrund der weiter in der Literatur verwendeten Bezeichnung wird auch hier der Begriff MODY für diese monogenetischen Diabetesformen verwendet.

### Epidemiologie

MODY gehört zu den seltenen Diabetesformen, eine häufig in der Literatur genannte Zahl beziffert die Prävalenz als < 5 % aller Diabetesfälle [4]. Die Prävalenz wird mit 1/23.000 bei Kindern und 1/10.000 bei jungen Erwachsenen angegeben [5–7]. Bei Kindern mit Diabetes liegt die Prävalenz zwischen 1,1 und 6 % mit hoher geografischer Variabilität. Laut Daten aus einem österreichischen Diabetesinzidenzregister für Kinder und Jugendliche sind 3,9 % aller Diabeteserkrankungen auf seltene Formen zurückzuführen, den größten Anteil davon machen genetische Formen (insbesondere Glucokinase-Mutationen und „hepatic nuclear factor 1A“[HNF1A]-Mutationen) und „cystic fibrosis related diabetes“ (CFRD) aus [8].

Ungeachtet der in Studien berichteten Prävalenz ist eine hohe Dunkelziffer im Sinne einer Unterdiagnose anzunehmen [5, 6, 9]. Studien aus Großbritannien legen nahe, dass mehr als 80 % der Fälle nicht diagnostiziert werden [5]. Als Gründe hierfür werden mangelndes Bewusstsein und Kenntnis zu MODY und ungleichgewichtiger Zugang zu genetischer Testung genannt. Jeder 8. im jugendlichen Erwachsenenalter diagnostizierte Diabetesfall (13 %) war nicht als klassischer Typ-1- oder Typ-2-Diabetes einzuordnen, demnach ein MODY anzunehmen [9]. Wird MODY nicht als solcher erkannt, resultiert häufig eine Fehlbehandlung [6].

Von den derzeit bekannten MODY-Genen betrifft die überwiegende Mehrzahl (etwa 95 %) aller Fälle Mutationen in lediglich 3 Genen, nämlich *HNF1A* (hepatic nuclear factor 1A), *HNF4A* und *GCK* (Glucokinase) [4, 10]. Die Kenntnis des Vorgehens bei Vorliegen einer dieser 3 Mutationen ist für klinische Belange überaus bedeutsam.

### Präzisionsmedizin

Der MODY – monogenetischer Diabetes – hat in den letzten Jahren auch im Kontext der Präzisionsmedizin an Aufmerksamkeit gewonnen, verstanden als maßgeschneiderte, individuelle Therapie für Patient:innen mit Diabetes, möglichst basierend auf der dem Diabetes zugrunde liegenden Pathophysiologie [11]. Die häufigsten heute bekannten MODY-Formen beruhen entweder auf Mutationen von B‑Zell-Transkriptionsfaktoren (HNF 1A und HNF 4A), wodurch die Insulinsekretion kompromittiert wird, oder auf einer Pathologie des die Freisetzung von Insulin auslösenden Glukoseschwellwertes (GCK). Aus den jeweiligen Mutationen resultierende Phänotypen sind klinisch ausreichend gut beschrieben und erlauben somit eine differenzialtherapeutische Zuordnung der Betroffenen [4]. Dies ist umso wichtiger, da die Möglichkeiten der Pharmakotherapie des Diabetes in den letzten Jahren deutlich zugenommen haben, wodurch die individuelle Therapieentscheidung generell deutlich komplexer geworden ist.

### Diagnostik

Aufgrund der unterschiedlichen Behandlungsansätze, die für die verschiedenen Subtypen des monogenetischen Diabetes verfügbar sind, wird der Bedeutung der Diagnosestellung des monogenetischen Diabetes zunehmend mehr Beachtung geschenkt. Eine Diagnose kann nicht nur eine Therapieumstellung auf eine orale Medikation anstatt einer Insulintherapie bedeuten, sondern auch familiäre Implikationen haben [12].

Die Verdachtsdiagnose MODY erfolgt auf der Grundlage klinischer Kriterien (Tab. 2). Aufgrund des autosomal-dominanten Erbganges sind Diabetesfälle in der Familie zu erwarten, insbesondere bei erstgradigen Verwandten, die oft über mehrere Generationen rückverfolgbar sind. Die Diagnose erfolgt häufig bereits im Kindes- und jungen Erwachsenenalter. Eine Diabetesdiagnose vor Vollendung des 30. Lebensjahres ohne Nachweis von beta-zellspezifischen Antikörpern oder ohne Notwendigkeit einer Insulintherapie (Differenzialdiagnose Typ-1-Diabetes) sollte an einen MODY-Diabetes denken lassen. Phänotypisch handelt es sich in der Regel um normalgewichtige Patient:innen (Differenzialdiagnose Typ-2-Diabetes).

**Tab. 2:** MODY/monogenetischer Diabetes: klinische Charakteristika. (Adaptiert nach [4])

I) Nachweis einer *transienten, neonatalen hyperinsulinämischen Hypoglykämie* (HNF-4A)
II) *Familienanamnese*, dem autosomal-dominanten Erbgang entsprechend. Davon zu unterscheiden: a) Typ-1-Diabetes tritt oft sporadisch auf; (lediglich) 2–6 % der Betroffenen haben einen ebenso betroffenen Elternteil. b) Typ-2-Diabetes tritt zwar auch familiär gehäuft auf, was allerdings dem meist ähnlichen Lebensstil als auch gemeinsamen Diabetesrisikoallelen geschuldet ist
III) *Frühmanifestierter Diabetes* (vor dem 35. Lebensjahr; vor dem 25. Lebensjahr mit höherer Wahrscheinlichkeit), wobei Folgendes nicht typisch für einen Typ-1-Diabetes ist:
a) Pankreatische Autoantikörper sind nicht nachweisbar, b) bei Insulintherapie ist eine meist nur geringe Insulindosis (z. B. < 0,5 IE/kgKG und Tag) erforderlich, c) C-Peptid (> 0,6 ng/ml) ist auch nach längerem Krankheitsverlauf (3 bis 5 Jahre) messbar, d) keine Ketoazidose
IV) *Untypisch für Typ-2-Diabetes:*
a) normaler BMI
b) keine Zeichen eines metabolischen Syndroms (normale Triglyceride, normales oder hohes HDL-Cholesterin) und
c) keine Zeichen einer Acanthosis nigricans und
d) milde, stabile Hyperglykämie ohne Zeichen einer Progression (GCK)
V) *Gutes Ansprechen auf Sulfonylharnstoffe*

Jedoch sind Diabeteserstmanifestationen bei MODY auch später möglich. In einer rezenten Studie aus UK und den USA zeigte sich bei Patient:innen mit einer Erstdiagnose Diabetes nach dem 40. Lebensjahr eine Prävalenz von MODY zwischen 0,25 und 1,1 %. Bis zu 50 % dieser Patient:innen erhielten eine nicht adäquate Therapie [13].

Das differenzialdiagnostische Prozedere umfasst die Bestimmung des Nüchternblutzuckers, C‑Peptids (außerhalb der Remissionsphase), HbA_1c_ sowie der bei Autoimmundiabetes nachweisbaren Antikörper (GAD, IA2, ZnT8, Insulinautoantikörper). Der Nachweis von Autoantikörpern macht das Vorliegen eines MODY sehr unwahrscheinlich, wenngleich Fälle eines koexistenten Typ-1-Diabetes beschrieben sind [14]. Ein MODY „clinical risk calculator“ ist online verfügbar (https://www.diabetesgenes.org/exeter-diabetes- app/), der die Wahrscheinlichkeit für das Vorliegen eines MODY unter Berücksichtigung klinischer Parameter zur Selektion von Patient:innen für die molekulargenetische Diagnostik errechnet [15].

Die Diagnose MODY kann – gewissermaßen retrospektiv – auch nach langem Diabetesverlauf gestellt werden. Die korrekte Diabetes-(Differenzial‑)Diagnose kann einerseits für Betroffene klinisch und therapeutisch (insbesondere gegenüber dem „klassischen“ Typ-2-Diabetes) relevant sein, andererseits auch für die direkte Nachkommenschaft im Sinne des autosomal-dominanten Erbganges.

Eine portugiesische Studie unter 73 Menschen (jünger als 35 Jahre) mit manifestem Diabetes, positiver Familienanamnese hinsichtlich Diabetes und negativen spezifischen Autoantikörpern zeigte bei 52 % einen genetischen MODY-Nachweis. Diese Studie beschreibt auch einen Wert von 36 % gemäß MODY-Calculator als hinweisenden Cut-off, um eine molekulargenetische Untersuchung in die Wege zu leiten [16].

Bei klinischem und anamnestischem Verdacht auf das Vorliegen eines MODY (s. Tab. 2) erfolgt die Diagnosesicherung mittels Genotypisierung. Diese erfolgt für klinische Belange unter Verwendung kommerziell verfügbarer NGS(„next generation sequencing“)-Plattformen. Sie ermöglichen die simultane und demnach mittlerweile vergleichsweise kostengünstige DNA-Analyse bekannter MODY-assoziierter Gene [17].

### Therapie

Für praktische Belange empfiehlt sich die Unterscheidung zwischen MODY-Formen, die einerseits Mutationen Beta-Zell-spezifischer Transkriptionsproteine (z. B. HNF) betreffen, sowie andererseits Mutationen im Glucokinase(*GCK*)-Gen.

### HNF-Mutationen: Erstlinientherapie

Sulfonylharnstoffe werden bei HNF-MODY-Formen generell als Erstlinientherapie empfohlen [1, 4, 10, 11].

### HNF1A-MODY

Bei Patient:innen mit HNF1A-MODY war der Sulfonylharnstoff Gliclazid um den Faktor 5 wirksamer als Metformin und um den Faktor 4 wirksamer als bei klassischem Typ-2-Diabetes [18]. Selbst bei Patient:innen, die bereits über lange Zeit (Median 20 Jahre) eine Insulintherapie erhalten hatten (in einer Dosierung von Median 0,5 IE/kgKG und Tag), erbrachte die Umstellung auf 80 mg/Tag Gliclazid eine HbA_1c_-Reduktion von 0,8 % [19]. Aufgrund der erhöhten Sensitivität der β‑Zellen für Sulfonylharnstoffe bei erhaltener (hoher) Insulinsensitivität wird eine kleine Startdosis empfohlen. Diese reicht oft aus, den gewünschten Therapieeffekt zu erzielen; das Risiko von Hypoglykämien ist vermutlich höher als beim klassischen Typ-2-Diabetes. Die Gabe von Nateglinid führte verglichen mit Glibenclamid zu abgeschwächten postprandialen Blutzuckerspitzen und zu einem geringeren Risiko von Hypoglykämien [20].

### HNF4A-MODY

Das Ansprechen auf Sulfonylharnstoffe ist bei HNF4A-Mutationen geringer ausgeprägt als bei HNF1A-MODY, sodass hier eher mit einem β‑Zell-Versagen und dann notwendiger Einleitung einer Insulintherapie zu rechnen ist [1].

### HNF-Mutationen: Zweitlinientherapie und experimentelle Ansätze

Bei langer Krankheitsdauer kann es auch bei MODY zur nachlassenden Wirksamkeit der Sulfonylharnstoffe kommen. Es wird angenommen, dass die Glukose-induzierte pankreatische Insulinsekretion ungeachtet fortgesetzter Sulfonylharnstoffgabe mit einer Rate zwischen 1 % und 4 % pro Jahr abfällt [1, 4]. Bei vielen Patient:innen mit HNF-MODY wird daher nach langem Verlauf die Einleitung einer Insulintherapie erforderlich sein, insbesondere bei HNF4A-Mutationen.

### Inkretin-basierte Therapien

Klinische Studien haben jüngst die Wirksamkeit von DPP-IV-Hemmern und GLP-1-Rezeptoragonisten bei MODY (HNF1A) untersucht mit dem Ziel, die β‑Zell-Funktion über einen längeren Zeitraum zu erhalten [21, 22]. Die Gabe von Linagliptin zu Glimepirid verbesserte bei 19 Patient:innen mit MODY-HNF1A gegenüber Placebo sowohl die Glukosevariabilität als auch den mittleren Blutzucker (HbA_1c_ minus 0,5 % bei einem Ausgangswert von 7,4 %), ohne dabei das Risiko von Hypoglykämien zu erhöhen [23]. Weiters war bei 16 Patient:innen der GLP-1-Rezeptoragonist Liraglutid dem Sulfonylharnstoff Glimeprid weitgehend ebenbürtig hinsichtlich glykämischer Kontrolle, bei geringerem Risiko von (milden) Hypoglykämien [24].

### SGLT-2-Hemmer

Der Einsatz von SGLT-2-Hemmern wird bei MODY nicht empfohlen [4]; das Risiko von Komplikationen wie höhergradige Dehydratation und DKA (diabetische Ketoazidose) [25] wird nach heutigem Wissensstand höher gewertet als der potenzielle Nutzen.

### Glucokinase(GCK)-Mutationen

Glucokinase-Mutationen stellen mit einer Häufigkeit von 0,1 % in der Allgemeinbevölkerung die häufigste MODY-Form dar [26]. Betroffene weisen typischerweise milde, stabile Erhöhungen des Nüchternblutzuckers auf. HbA_1c_-Werte können erhöht sein, typischerweise unter 7,5 % [10]. GCK-MODY wird bei Erwachsenen oft fälschlicherweise als Typ-2-Diabetes klassifiziert, im jüngeren Lebensalter folgt die Diagnose Diabetes häufig einer aus anderen Gründen veranlassten Blutanalyse im Sinne eines Zufallsbefundes. Mit der weiteren Verbreitung von Diabetesfrüherkennungsprogrammen (wie etwa 2021 die in Österreich erfolgte Kostenerstattung der HbA_1c_-Messung) ist damit zu rechnen, dass GCK-Mutationen häufiger detektiert werden. Daher ist die Kenntnis des GCK-MODY von großer Bedeutung; ebenso die korrekte differenzialdiagnostische Zuordnung (inklusive Genotypisierung), einerseits um Betroffene entsprechend aufklären zu können (hinsichtlich Erbgang, Krankheitsverlauf, Implikationen für die medikamentöse Behandlung, Implikationen für etwaige Schwangerschaften), andererseits um unnötige Pharmakotherapien hintanzustellen. Patient:innen mit GCK-MODY bedürfen außer einer entsprechenden Lebensstilmodifikation prinzipiell *keiner* spezifischen, blutzuckersenkenden Therapie [1, 4, 10, 11, 27], es sei denn, es liegt konkomitant ein Typ-1/Autoimmundiabetes [14] oder ein Typ-2-Diabetes vor oder im Falle einer Schwangerschaft (s. unten). Diese Empfehlung beruht auf Langzeitstudien, wonach Betroffene – wohl aufgrund der persistierend lediglich milden Hyperglykämie – keine diabetestypischen Komplikationen entwickeln [27–30].

### Schwangerschaft bei GCK-MODY

Wenn nicht ohnehin ein GCK-MODY präkonzeptionell bekannt ist, wird bei betroffenen Schwangeren regelhaft ein Gestationsdiabetes diagnostiziert werden, weil ein durchgeführter Glukosebelastungstest (oGTT) eine Pathologie ausweist (typischerweise einen erhöhten Nüchternblutzucker).

Die Betreuung von Schwangeren mit GCK-MODY, insbesondere die Notwendigkeit der Gabe von Insulin, hängt davon ab, ob der Fetus ebenfalls Träger der Mutation ist oder nicht [4, 31, 32]. Dies ist aus praktischen Belangen am ehesten aus der fetalen Biometrie abzuleiten. Wenn die Variante beim Fetus nicht vorliegt (oder der Verlauf dies nahelegt), besteht ein hohes Makrosomierisiko. Ob in diesem Fall eine Insulintherapie indiziert ist, wird heftig diskutiert, da diese hohe Dosen erfordert und mit einem sehr hohen Hypoglykämierisiko einhergeht [31]. In vielen Fällen wird man sich für eine engmaschige Biometrie und eine vorgezogene Entbindung entscheiden. Jedenfalls gehören diese Patient:innen in die interdisziplinäre (Endokrinologie, Gynäkologie) Betreuung eines Zentrums.

Im Gegensatz dazu, bei Vorliegen einer Mutation die durch den Vater vererbt wurde, besteht aufgrund der vergleichsweise niedrigen Insulinspiegel bei Euglykämie der Mutter das Risiko für eine fetale Dystrophie und Wachstumsretardierung [33].

### HNF-1β-Mutation

Eine Sonderstellung unter den MODY-Formen nimmt der HNF1B-MODY (Typ 5) aufgrund der möglichen extrapankreatischen Manifestationen mit Nierenerkrankungen, Fehlbildungen im Genitaltrakt, erhöhten Leberwerten, Magnesiummangel oder neurologischen Auffälligkeiten ein [34, 35].

### Seltenere MODY-Formen

Es sind derzeit Mutationen in mehr als 15 MODY-Genen beschrieben [4], wobei die Liste eine ständige Erweiterung erfährt. Oft erleichtert auch bei diesen selteneren Formen die der Mutation folgende Pathologie die Therapieentscheidung; häufig ist (analog den HNF-Mutationen) die β‑Zelle betroffen, und Sulfonylharnstoffe gelten demnach als Therapie der ersten Wahl. Allerdings ist bei der Interpretation von molekulargenetischen Befunden zu beachten, dass für manche (eben seltene) Mutationen, die im Zusammenhang mit MODY genannt werden, die tatsächliche, klinisch relevante Pathogenität noch geklärt werden muss. Dies gilt etwa für Mutationen von BLK, KLF11, NEUROD1, PAX4, PDX1, die zum Teil in der Allgemeinbevölkerung so häufig auftreten, dass der Zusammenhang mit MODY nicht gesichert ist [4]. Bei Diagnose einer dieser seltenen MODY-Formen wird daher die Zuweisung an spezialisierte Zentren empfohlen.

### Mitochondriale Diabetesformen

Mitochondriale Diabetesformen sind – sofern es sich um keine Spontanmutation handelt – maternal vererbt und häufig mit neuromuskulären Symptomen sowie Hörstörungen verbunden, wobei Letztere häufig der Diabetesmanifestation vorausgehen. Laut Diabetes-Patient:innen-Verlaufsdokumentation (DPV)-Register machen diese Formen weniger als 1 % aller Diabetesfälle im Erwachsenenalter aus [36]. Den beiden Syndromen „maternally inherited diabetes and deafness“ (MIDD) und „mitochondrial encephalomyopathy with lactic acidosis and stroke-like episodes“ (MELAS) liegt die gleiche Genmutation (m.3243A > G) zugrunde [37]. Vermutlich aufgrund der verschieden ausgeprägten Heteroplasmie kommt es jedoch zu unterschiedlicher Symptomatik [38]. Das DIDMOAD- oder Wolfram-Syndrom ist charakterisiert durch das Auftreten eines Diabetes insipidus, eines Diabetes mellitus, einer Optikusatrophie und Innenohrschwerhörigkeit bis zur Taubheit. Eine spezifische Therapie der mitochondrialen Diabetesformen existiert nicht, beim MELAS-Syndrom werden L‑Arginin und auch Carnitin bzw. Coenzym Q10 zur Therapie der neurologischen Symptomatik versuchsweise eingesetzt [39]. Beim Kearns-Sayre-Syndrom handelt es sich um eine mitochondriale Diabetesform, die auch durch eine Augen- und Herzbeteiligung gekennzeichnet ist [40].

### Neonataler Diabetes (NDM)

Der neonatale Diabetes ist eine seltene Form des monogenetischen Diabetes und definiert als Diabetes mit Manifestation vor dem 6. Lebensmonat, wobei transiente von permanenten Verläufen unterschieden werden. Ein transienter neonataler Diabetes ist durch eine Normalisierung des Glukosestoffwechsels bis zum 18. Lebensmonat definiert. Wenn auch seltener, können monogene Formen des NDM auch bis zum 12. Lebensmonat auftreten [41]. Ein Diabetesrelaps bei transienten Verlaufsformen wird in bis zu 50 % der Fälle beschrieben [42]. In Österreich beläuft sich die Inzidenz auf 1/230.000 für transiente und 1/530.000 für permanente neonatale Diabetesformen [43]. Die Ursache ist meist monogenetisch, wobei Mutationen an Untereinheiten des ATP-abhängigen Kaliumkanals (*ABCC8, KCNJ11*) in 40 % ursächlich für einen neonatalen Diabetes sind. 6q24-Abnormitäten (uniparentale Disomie, Duplikation, Methylierungsdefekte) sowie Mutationen am Proinsulin codierenden *INS*-Gen, Glucokinase-Gen *GCK, HNF1β* sind ebenso ursächlich beschrieben [44]. Ein neonataler Diabetes kann zudem auch im Rahmen syndromaler Erkrankungen auftreten, wobei das IPEX-Syndrom (*FOXP3*), das Wolcott-Rallison-Syndrom (*EIF2AK3*) und die Pankreasagenesie (*PDX1, PTF1A*) ca. 10 % der neonatalen Diabetesfälle erklären. Zudem ist das Auftreten eines neonatalen Diabetes im Rahmen seltener Mutationen in Genen, die für die Immunfunktion wichtig sind (*FOXP3, LRBA, STAT1, STAT3*), beschrieben.

Ein NDM sollte bei einer Laborkonstellation mit vermindertem C‑Peptid und Insulin sowie erhöhten Ketonen immer bei Säuglingen mit insulinabhängiger Hyperglykämie in Betracht gezogen werden, wenn die Blutzuckerwerte kontinuierlich über 250 mg/dl liegen und keine andere Ursache der Hyperglykämie vorliegt. Die genetische Abklärung ist aufgrund therapeutischer Konsequenzen wichtig [45, 46]. Ebenso sollte eine Pankreassonographie erfolgen, da diese die Therapieentscheidung beeinflusst. Eine Antikörperdiagnostik ist vor dem 6. Lebensmonat nur bedingt aussagekräftig.

Die initiale Behandlung mit Insulin unter Vermeidung von Hypoglykämien ist immer indiziert. Aufgrund der häufig erforderlichen nur sehr geringen Insulindosen, der fehlenden Präzision von Pens sowie der erschwerten Kohlenhydratberechnung bei ad libidum gestillten Kindern findet die Verwendung einer Insulinpumpe oft auch mit verdünntem Insulin (U10, U50) bevorzugt Einsatz. Auch der „off-label use“ einer sensorunterstützen Pumpentherapie im Hybrid-closed-loop-Modus ist bei ausreichend subkutanem Fettgewebe ab einem Körpergewicht von ca. 3 kg möglich. Eine orale Behandlung mit Sulfonylharnstoffen ist indiziert, wenn Mutationen an den Untereinheiten SUR1 und Kir6.2 des ATP-abhängigen Kaliumkanals als Ursache für den vorliegenden neonatalen Diabetes genetisch bestätigt wurden [47]. Hier stehen auch orale Suspensionen zur Therapie bei Säuglingen zur Verfügung [48].

## II) Diabetesformen im Rahmen anderer endokriner Erkrankungen

Endokrinologische Erkrankungen, die mit Glukosestoffwechselstörungen einhergehen, und deren diagnostische Tests sind in Tab. 3 dargestellt.

**Tab. 3:** Endokrinologische Erkrankungen mit erhöhtem Risiko für das Auftreten einer Glukosetoleranzstörung sowie empfohlene Screeninguntersuchungen bzw. diagnostische Tests. (Adaptiert nach [86, 138])

Erkrankung	Screening und Diagnostik
Cushing-Syndrom	24 h-Harn-Cortisol, Serum- oder Speichel-Mitternachtscortisol, 1 mg Dexamethason-Suppressionstest
Akromegalie	Serum-IGF‑1, 75 g OGTT: GH Nadir
Hyperthyreose	TSH, fT4, fT3
Phäochromozytom	Serum- oder Harn-Metanephrin/Normetanephrin
Primärer Hyperaldosteronismus	Aldosteron-Renin-Quotient
Glukagonom	Plasma-Glukagon
Somatostatinom	Plasma-Somatostatin

*IGF‑1* „insulin-like growth factor 1“, *OGTT* oraler Glukosetoleranztest, *TSH* Thyreotropin

### Cushing-Syndrom

In der überwiegenden Mehrzahl ist ein Cushing-Syndrom auf eine vermehrte ACTH (Adrenocorticotropin)-Produktion zurückzuführen. Diese wiederum tritt am häufigsten bei ACTH-sezernierenden Hypophysenadenomen auf, seltener sind ektope ACTH-Quellen bei meist malignen Tumoren (z. B. Bronchialkarzinom, Thymuskarzinoid). Die häufigste Ursache für ACTH-unabhängige Cushing-Syndrome sind Nebennierenadenome, seltener Nebennierenkarzinome oder mikro- oder makronoduläre adrenale Hyperplasien [49]. Die klinischen Symptome ähneln teils den diagnostischen Kriterien eines metabolischen Syndroms. Neben einer Gewichtszunahme, einer gestörten Glukosetoleranz bis zum Auftreten eines Diabetes mellitus und einer arteriellen Hypertonie finden sich häufig osteoporotische Wirbelkörperfrakturen, ein Hypogonadismus und Hautveränderungen, insbesondere rote Striae (Striae rubrae), eine Myopathie und auch psychiatrische Auffälligkeiten [49]. Die Therapie erfolgt, sofern möglich, chirurgisch, je nach Ätiologie finden ansonsten Dopaminagonisten oder das Somatostatinanalogon Pasireotid (Hypophysenadenome) oder adrenostatisch bzw. adrenolytisch wirkende Medikamente (ektope oder adrenale Formen) bzw. strahlentherapeutische Verfahren Anwendung (hypophysäre Formen) [49]. Bezüglich therapeutischer Überlegungen s. medikamentös induzierte Diabetesformen.

### Akromegalie

Zum ganz überwiegenden Teil ist eine überschießende Sekretion von Wachstumshormon bei Hypophysenadenomen Ursache einer Akromegalie, nur sehr selten ist eine ektope GHRH-Sekretion für eine Akromegalie verantwortlich. Neben unspezifischen Symptomen wie Cephalea, Hyperhidrosis, Schlafapnoe, Hypertonie und Arthralgien sind akrales Wachstum und Makroglossie charakteristische Symptome einer Akromegalie. Begleitet werden diese von metabolischen Komplikationen in Form einer Glukosetoleranzstörung bzw. eines manifesten Diabetes mellitus sowie einer Dyslipidämie [50]. Die Serum-„Insulin-like growth factor 1“(IGF-1)-Konzentration wird zum Screening herangezogen, wobei ein präexistenter Diabetes mellitus ebenso wie katabole Zustandsbilder oder eine eingeschränkte Leberfunktion zu falsch negativen Ergebnissen führen können. Bestätigt wird das Vorliegen einer Akromegalie mittels eines 75 g oralen Glukosetoleranztests (OGTT) (GH Nadir > 0,4 µg/l). Bei unklaren diagnostischen Konstellationen kann bei Patient:innen mit diabetischer Stoffwechsellage ein dynamischer Test mit Galanin oder Thyreotropin-Releasing-Hormon erwogen werden [51].

Therapeutisch ist eine chirurgische Sanierung anzustreben, sollte diese nicht möglich bzw. nicht erfolgreich sein, kommen bei milden Formen Dopaminagonisten, üblicherweise jedoch Somatostatinanaloga oder der GH-Rezeptor-Antagonist Pegvisomant zum Einsatz, ggf. auch strahlentherapeutische Verfahren [50, 52].

Pathophysiologisch führt eine kontinuierliche exzessive Erhöhung der GH-Konzentration zu einer Störung im Insulinsignaltransduktionsweg, klinisch einer Insulinresistenz entsprechend. Die Therapie eines Cushing- oder Akromegalie-assoziierten Diabetes unterscheidet sich prinzipiell nicht von der eines Diabetes mellitus Typ 2 [51]. Die Behandlung der Grunderkrankung führt bei Patient:innen mit Cushing- oder Akromegalie-assoziiertem Diabetes meist zu einer Verbesserung der glykämischen Kontrolle. Hier bildet lediglich die Therapie mittels Pasireotid eine Ausnahme. Pasireotid wird sowohl in der Therapie des Cushing als auch bei Akromegalie eingesetzt. Neben einer Suppression von ACTH und GH kommt es auch zu suppressiven Effekten auf GLP‑1, „glucose-dependent insulinotropic polypeptide“ (GIP) und Insulin, sodass unter der Therapie mittels Pasireotid häufig eine Verschlechterung der glykämischen Kontrolle beobachtet werden kann [53, 54].

### Andere mit DM assoziierte Endokrinopathien

Ein primärer Hyperaldosteronismus, ebenso wie ein Phäochromozytom, eine manifeste Hyperthyreose oder die seltenen neuroendokrinen Neoplasien Glukagonom und Somatostatinom können mit Störungen des Glukosestoffwechsels einhergehen (Tab. 3).

## III) Medikamentös induzierte Diabetesformen

Zahlreiche Medikamente führen in unterschiedlicher Ausprägung zu einer Beeinträchtigung des Glukosestoffwechsels, wichtige Vertreter sind in Tab. 4 zusammengefasst. Im Folgenden wird detailliert auf Substanzen eingegangen, die entweder akute behandlungsbedürftige Hyperglykämien hervorrufen können oder mit einem deutlich erhöhten Diabetesrisiko einhergehen.

**Tab. 4:** Medikamente, für die in unterschiedlicher Ausprägung eine Beeinträchtigung des Glukosestoffwechsels beschrieben wurde [86, 138–148]

Hormone	Glukokortikoide
	Somatotropin, Tesamorelin
	„Gonadotropin-releasing hormone agonists“
Onkologische Therapeutika	„Programmed cell death receptor 1 (PD1) inhibitors“ (e. g. Nivolumab, Pembrolizumab, Cemiplimab), „Programmed cell death ligand 1“(PD-L1)-Inhibitoren (e. g. Atezolizumab, Avelumab, Durvalumab)
	„Cytotoxic T‑lymphocyte associated protein 3“(CTL4)-Inhibitoren (z. B. Ipilimumab, Tremelimumab)
	PI3K-Inhibitoren (z. B. Alpelisib)
Antipsychotika	Erstgenerationsantipsychotika (Chlorpromazin, Perphenazin, andere Phenothiazine)
	Zweitgenerationsantipsychotika (Clozapin, Iloperidon, Olanzapin, Paliperidon, Quetiapin, Risperidon)
Immunsuppressiva	Tacrolimus, Cyclosporin A, Sirolimus, Glukokortikoide
HAART	Antiretrovirale HIV-Therapie (Proteaseinhibitoren, nukleosidische Reverse-Transkriptase-Inhibitoren [RTIs])
Vasopressoren	Adrenalin, Noradrenalin
Lipidsenker	Nikotinsäurederivate, Statine
Antihypertensiva	Betablocker (Atenolol, Metoprolol, Propranolol)
	Thiaziddiuretika (Hydrochlorothiazid, Chlorthalidon, Chlorothiazid, Indapamid)
Andere	Somatostatinanaloga, Diazoxid, Interferon‑γ, Pentamidin, Fluorchinolone (Moxifloxacin, Gatifloxacin)

### Glukokortikoide und andere Immunsuppressiva

Anstatt des häufig verwendeten Terminus Steroid-induzierter Diabetes sollte besser, weil präziser, Glukokortikoid-induzierter Diabetes Anwendung finden. Jegliche systemische Glukokortikoidtherapie ist mit einer erhöhten Diabetesinzidenz verbunden. Eine nur kurzzeitige, meist hoch dosierte Glukokortikoidtherapie kann zu einer transienten Hyperglykämie führen, während eine länger dauernde Glukokortikoidtherapie u. a. durch eine Gewichtszunahme auch zu einer persistierenden Hyperglykämie führen kann [55]. Die Dosis des Glukokortikoids, bei welcher eine signifikante Blutzuckererhöhung zu erwarten ist, hängt von den individuellen Risikofaktoren ab und steigt ab einem Methylprednisolonäquivalent von 4 mg deutlich an [56]. Topische oder inhalative Glukokortikoide führen in der Regel zu keiner nennenswerten Blutzuckererhöhung. Auch ein bereits bestehender Diabetes mellitus kann durch eine Glukokortikoidtherapie aggraviert werden. Das Risiko für das Auftreten einer Hyperglykämie ist vor allem bei > 65-jährigen Patient:innen, zugrunde liegender Glukosestoffwechselstörung, reduzierter Nierenfunktion und bei Patient:innen mit rheumatologischen bzw. nephrologischen Erkrankungen in Abhängigkeit von der Glukokortikoiddosis und Therapiedauer signifikant erhöht [57, 58]. Charakterisiert ist ein Glukokortikoid-induzierter Diabetes bei morgendlicher Glukokortikoidgabe durch eine meist normale Nüchternglukosekonzentration und eine ausgeprägte Hyperglykämie untertags, insbesondere wenn intermediär wirksame Glukokortikoide (z. B. Prednisolon oder Methylprednisolon) eingesetzt werden. Infolgedessen sind postprandiale Glukosemessungen zum Screening und zur Monitorisierung besser geeignet als alleinige Nüchternglukosemessungen [59]. Bei länger wirksamen Glukokortikoiden (z. B. Dexamethason) kann die Hyperglykämie bis zum Folgetag persistieren. Der HbA_1c_-Wert eignet sich nur bei längerfristiger chronischer Glukokortikoidtherapie zur Therapieüberwachung.

Die diabetogene Stoffwechsellage ist bei Hyperkortisolismus neben einer meist ausgeprägten Gewichtszunahme auch auf eine hepatische und periphere Insulinresistenz sowie eine verminderte Insulinsekretion zurückzuführen [60]. Die Behandlung unterscheidet sich nicht von der eines Diabetes mellitus Typ 2 [61]. Sie richtet sich nach Pharmakokinetik des verwendeten Glukokortikoidpräparates und dem daraus resultierenden Blutzuckertagesprofil sowie den Komorbiditäten der Patient:innen. Als Ziel, insbesondere im stationären Bereich, gilt es, einen Blutzucker von < 180 mg/dl zu erreichen. Problematisch stellt sich dar, dass Menschen, die Glukokortikoide benötigen, meist eine vulnerable Patient:innenpopulation darstellen (z. B. Zustand nach Organtransplantationen, hämato(onkologische Patient:innen)). Insbesondere bei akut kranken oder hospitalisierten Menschen ist eine Insulintherapie die sicherste Methode zur Blutzuckersenkung. Generell ist die Evidenz zu oralen antidiabetischen Therapeutika bei Glukokortikoid-induziertem Diabetes sehr gering. Der Einsatz von DPP4(Dipeptidyl-peptidase IV)-Hemmern oder Sulfonylharnstoffen bei milden Hyperglykämien ist hierbei noch am besten mit Evidenz belegt [61].

Eine besondere Diabetesform stellt der sog. Posttransplantationsdiabetes („new onset of diabetes after transplantation“ [NODAT]) dar. Die Prävalenz wird abhängig von dem transplantierten Organ langfristig mit bis zu 40 % angegeben [62, 63]. Neben Glukokortikoiden weisen vor allem Calcineurininhibitoren (insbesondere Tacrolimus) ein diabetogenes Potenzial auf, da sie gleichzeitig zu einer Verschlechterung der Insulinsensitivität und der Insulinsekretion führen können [64]. Die Behandlung des NODAT unterscheidet sich zwar grundsätzlich nicht von der eines Diabetes mellitus Typ 2, die Therapieoptionen sind jedoch häufig aufgrund der Komorbiditäten eingeschränkt.

### Onkologische Therapien

Sowohl Immuncheckpointinhibitoren (ICI) als auch Phosphatidylinositol-3-kinase-Inhibitoren können akute Hyperglykämien auslösen, wenn auch auf unterschiedlicher Basis.

Immuncheckpointinhibitoren, insbesondere „programmed cell death“(PD)-1 bzw. PD-Ligand-1-Inhibitoren wie Pembrolizumab oder Nivolumab, seltener auch CTLA-4-Inhibitoren wie Ipilimumab, führen bei bis zu 1,9 % der behandelten Patient:innen zu einer akuten Hyperglykämie. Diese ist auf eine Destruktion der Betazellen zurückzuführen, wobei bei weniger als 50 % der Betroffenen spezifische Antikörper nachweisbar sind. Die Beeinträchtigung des Kohlenhydratstoffwechsels tritt meist innerhalb der ersten 3 Behandlungsmonate auf, häufig ist eine diabetische Ketoazidose die Erstmanifestation. Das HbA_1c_ ist aufgrund des akuten Auftretens nicht aussagekräftig. Vier- bis 6‑wöchentlich sollten – optimalerweise vor jedem Therapiezyklus – morgendliche Serumglukosewerte bestimmt werden. Aufgrund der Betazelldestruktion ist bei Manifestation eines Diabetes meist eine funktionelle Insulintherapie indiziert. Bei Patient:innen mit präexistentem DM 2 kann es zu einer Verschlechterung der Glykämie kommen [65, 66].

Phosphoinositid-3-Kinasen(PI3K)-Inhibitoren (z. B. Alpelisib) führen durch direkte Interaktion mit dem intrazellulären Insulinsignaltransduktionsweg zu einer meist innerhalb von 2 Wochen auftretenden Verschlechterung der Insulinsensitivität und Hyperglykämie. Speziell bei Patient:innen mit Prädiabetes, Adipositas, höherem Alter oder präexistentem Diabetes wird schon vor Therapiestart eine Vorstellung beim/bei der EndokrinologIn/DiabetologIn empfohlen, eine präventive Metformin-Gabe wird zudem von manchen Expert:innen diskutiert [67]. In der SOLAR-1-Studie trat eine Hyperglykämie bei mehr als 50 % der Patient:innen mit Normoglykämie vor Therapiestart und Normalgewicht auf, sodass eine entsprechende Schulung auch bei Patient:innen ohne präexistente Risikofaktoren vorab erfolgen sollte [68]. Als Screening werden in Abhängigkeit vom individuellen Diabetesrisiko 1‑ bis 2‑wöchentliche Nüchternglukosebestimmungen und in weiterer Folge 3‑monatlich HbA_1c_-Bestimmungen empfohlen [67]. Nachdem PI3K-Inhibitoren beispielsweise bei Mammakarzinom speziell bei aktivierender PI3K-Mutation zum Einsatz kommen, besteht pathophysiologisch ein gewisser onkologischer Vorbehalt zur Insulintherapie, die den PI3K-Weg wieder aktiviert, daher sollen primär Metformin, Pioglitazon und SGLT-2-Inhibitoren zur Therapie einer Alpelisib-induzierten Hyperglykämie verwendet werden [67], wobei bei einer Pioglitazon-Therapie auf ein erhöhtes Osteoporoserisiko zu achten ist [69].

Andere Medikamente, die häufig mit einer Verschlechterung des Glukosestoffwechsels einhergehen:

HAART (highly active anti-retroviral therapy), insbesondere Proteaseinhibitoren und nukleosidische Reverse-Transkriptase-Inhibitoren (NRTIs), können gemeinsam mit der Inflammation einer chronischen HIV-Infektion diabetogen wirken. So zeigte eine rezente Arbeit an 522 österreichischen Patient:innen mit chronischer HIV-Infektion, dass 50 % der im Schnitt 42-jährigen Teilnehmer insulinresistent waren [70]. Zusätzlich zeigte sie ein geringes Bewusstsein für metabolische Erkrankungen bei der Therapie dieser vulnerablen Gruppen: 60 % der Diabetesfälle waren nicht diagnostiziert, nur 13 % der Patient:innen mit Dyslipidämie waren therapiert. Die Wahl metabolisch neutraler antiretroviraler Medikation, regelmäßige Kontrollen metabolischer Parameter und ggf. frühzeitige Therapie scheinen ratsam (s. bitte Absatz Insulinresistenz und Diabetes bei Lipodystrophiesyndromen).

Insbesondere verschiedene typische, aber auch atypische Antipsychotika – unter anderem bedingt durch eine gesteigerte Nahrungszufuhr und entsprechende Gewichtszunahme – sind mit einem erhöhten Diabetesrisiko verbunden (s. Kapitel Abrahamian et al., Psychische Erkrankungen und Diabetes mellitus) [71].

### Medikamente, die mit einer milden Verschlechterung des Glukosestoffwechsels einhergehen können

In der Gruppe der Antihypertensiva weisen vor allem Thiaziddiuretika und nicht vasodilatierend wirkende Betablocker ein mildes diabetogenes Potenzial auf [72]. In Metaanalysen wird wiederholt ein gering, aber signifikant erhöhtes Diabetesrisiko von Patient:innen mit Statin-Therapie beschrieben [73–75]. Im Schnitt ist der HbA_1c_-Wert bei Personen mit Diabetes um 0,14 % höher, der Effekt scheint bei Männern ausgeprägter zu sein als bei Frauen [76]. In einer rezenten großen Metaanalyse aus der CTT (Cholesterol Treatment Trialists Collaboration Group) fand man einen dosisabhängigen Effekt (RR 1,36 für neu diagnostizierten Diabetes mellitus [95 % CI 1,25–1,48] in der mit hochpotenten Statinen behandelten Gruppe), allerdings überwiegt der kardiovaskuläre Nutzen im Vergleich zum erhöhten Diabetesrisiko, und jedwede Risikoerhöhung durch neu aufgetretene Diabetesfälle wäre ohnehin in die jeweiligen primären Endpunkte zum kardiovaskulären Risiko eingeflossen [77].

## IV) Insulinresistenz und Diabetes bei Lipodystrophiesyndromen

Die Lipodystrophiesyndrome umfassen eine heterogene Gruppe von seltenen kongenitalen und erworbenen Erkrankungen, die durch einen generalisierten oder partiellen Verlust von Fettgewebe charakterisiert sind, der nicht auf Mangelernährung zurückzuführen ist [78–80]. Sie sind mit einer Reihe von metabolischen Komplikationen wie Insulinresistenz, Diabetes mellitus, Hypertriglyzeridämie und Steatosis hepatis vergesellschaftet, häufig auch mit Acanthosis nigricans, polyzystischem Ovarsyndrom und eruptiven Xanthomen. Die Ausprägung der Komorbiditäten variiert bei den verschiedenen Subtypen und ist zum Teil auf die organspezifische ektope Fettakkumulation vor allem in Leber und Muskulatur zurückzuführen. Hormone des Fettgewebes, allen voran Leptin, sind reduziert. Dies trägt zu den metabolischen Störungen und zur häufig assoziierten Hyperphagie bei.

Die 2 häufigsten genetischen Formen sind die autosomal-rezessiv vererbte kongenitale generalisierte Lipodystrophie und die autosomal-dominant vererbte familiäre partielle Lipodystrophie mit jeweils mehreren Subtypen [78–80]. Weitere Formen sind die erworbene generalisierte Lipodystrophie, die erworbene partielle Lipodystrophie und die Progerie-assoziierte Lipodystrophie. Die Prävalenz von genetischen Lipodystrophien wird auf 1/1.000.000 Menschen geschätzt, die Zahl der undiagnostizierten Erkrankungen ist hoch [78]. Die durch die antiretrovirale Therapie mit Proteaseinhibitoren und nukleosidischen Reverse-Transkriptase-Inhibitoren bei HIV-Patient:innen und Patient:innen induzierte Lipodystrophie ist die häufigste Variante der erworbenen Lipodystrophien, diese tritt mit neuen Therapien aber nun seltener auf [81].

Die Therapie hat die Besserung bzw. Prävention der mit Lipodystrophie assoziierten Komorbiditäten zum Ziel. Neben der Lebensstilmodifikation sind das in erster Linie Metformin und Fibrate, andere orale Antidiabetika und Insulin, Omega-3-Fettsäuren sowie das Leptin-Analogon Metreleptin [79, 80]. Darüber hinaus ist noch eine Reihe anderer Pharmaka insbesondere zur Reduktion der erhöhten Triglyzeridkonzentrationen in klinischer Erprobung [80].

## V) Pankreopriver Diabetes (Typ-3c-Diabetes)

Generell kann jede Erkrankung, die zu einer diffusen Zerstörung des Pankreasgewebes führt, mit einem pankreopriven („pancreatogenic“) Diabetes einhergehen. In westlichen Ländern sind pankreoprive Ursachen für etwa 5–10 % aller Diabeteserkrankungen verantwortlich [82]. Ätiologisch kommen dafür Pankreatitiden, Pankreaskarzinome, Pankreasoperationen und seltener Traumata, Infektionen oder Pankreasagenesie infrage. Pankreatitiden sind für etwa drei Viertel aller pankreopriven Diabetesformen verantwortlich [83]. Pankreaskarzinome stellen insofern eine Ausnahme dar, als sie auch bei lokalisiertem Vorliegen ohne diffuse Zerstörung des Pankreasgewebes zu einer diabetogenen Stoffwechsellage führen können, der Grund dafür ist bisher nicht klar [84]. Schätzungen zufolge liegt bei 85 % der mit Pankreaskarzinom diagnostizierten Patient:innen eine Glukosestoffwechselstörung und bei 50 % ein manifester Diabetes vor. Insofern ist bei Neuauftreten eines Diabetes bei entsprechendem Risikoprofil (Pankreatitis in der Anamnese, Alkoholabusus, Gewichtsabnahme, positive Familienanamnese) eine Bildgebung des Pankreas mittels hochauflösender Schnittbilddiagnostik zu empfehlen. Menschen mit Diabetes wird ein höheres Pankreaskarzinomrisiko im Vergleich zur Allgemeinpopulation zugeschrieben. Das Auftreten eines Diabetes nach einer Pankreasoperation scheint zudem mit einem höheren Mortalitätsrisiko assoziiert zu sein, wobei das Risiko bei Malignomen höher ist als bei Operationen aufgrund von nichtmalignen Pankreaserkrankungen [85]. Hereditäre Stoffwechselerkrankungen wie zystische Fibrose und Hämochromatose können ebenso zu pankreoprivem Diabetes führen. Auch die seltene und primär in asiatischen Ländern vorkommende fibrokalkulöse Pankreatopathie (FCPP) kann sekundär einen Diabetes mellitus bedingen [27, 86].

Nach Pankreasoperationen ist das Risiko für die Entwicklung eines pankreopriven Diabetes von Art und Ausmaß der Operation abhängig. Naturgemäß beträgt das Risiko 100 % bei total pankreatektomierten Patient:innen, bei Patient:innen mit Whipple Operation (partielle Pankreatikoduodenektomie) wird das Risiko in älteren Studien mit 26 % beziffert, wobei ein beträchtlicher Teil der damals untersuchten Patient:innen schon präoperativ an einem Diabetes erkrankt war [87].

Diagnostische Kriterien eines pankreopriven Diabetes umfassen eine Betazelldysfunktion, das Fehlen von Autoimmunantikörpern und das Vorliegen einer Erkrankung des exokrinen Pankreas (Diagnostik s. unten) [88, 89]. Die betroffenen Patient:innen weisen typischerweise eine hohe Insulinsensitivität auf, vorausgesetzt, dass dem pankreopriven Diabetes kein Diabetes mellitus Typ 2 oder Prädiabetes mit Insulinresistenz vorausgegangen ist.

Eine zufriedenstellende glykämische Kontrolle ohne Auftreten von klinisch signifikanten Hypoglykämien kann sich bei pankreoprivem Diabetes als schwierig herausstellen. Ursache dafür sind das Fehlen der gegenregulatorisch wirkenden Hormone Somatostatin und Glukagon ebenso wie eine durch die Grundkrankheit bedingte Malabsorption. Hinzu können schlechte Adhärenz und Lifestyle-Problematik bei Patient:innen mit alkoholischer Pankreatitis kommen [88].

Die systemische Insulinsensitivität ist bei pankreoprivem Diabetes typischerweise normal oder sogar erhöht, die hepatische Insulinsensitivität kann auch vermindert sein. Zur Therapie des pankreopriven Diabetes liegen keine spezifischen internationalen Richtlinien vor. Eine Metformin-Therapie kann bei gleichzeitig bestehender Insulinresistenz zu einer Verbesserung der Glykämie führen, allerdings wird Metformin aufgrund der gastrointestinalen Nebenwirkungen von diesem Patient:innenkollektiv oft schlecht toleriert. Bei hoher Insulinsensitivität ist der Effekt auf die Glykämie zudem als begrenzt anzunehmen. Studien belegen ein deutlich vermindertes Pankreaskarzinomrisiko unter Metformin-Therapie [89]. Inkretin-basierte Therapien sollte aufgrund der nicht ausreichenden Datenlage in diesem Kollektiv nicht verwendet werden. Auch Sulfonylharnstoffe und Glinide stellen bei diesen Patient:innen aufgrund des beträchtlichen Hypoglykämierisikos keine sichere Therapieform dar. SGLT-2-Hemmer sind aufgrund des vorliegenden Insulinsekretionsdefizits und des damit verbunden erhöhten Risikos einer euglykämischen Ketoazidose ebenfalls nicht zu empfehlen. Therapie der Wahl ist daher bei einem Großteil der betroffenen Patient:innen Insulin, optimalerweise in Form einer funktionellen Insulintherapie. Vereinzelte Praxiserfahrungen legen sehr positive Effekte von AID(automated insulin delivery)-Systemen nahe, randomisierte, prospektive Studien hierfür sind derzeit aber nicht vorhanden. In einer Studie konnte eine Überlegenheit eines Closed-loop-Systems gegenüber einer Standardinsulintherapie bei Patient:innen, die sich einer partiellen oder totalen Pankreatektomie unterziehen mussten, gezeigt werden [90]. Aufgrund der dynamischen Vorausberechnung des Insulinbedarfs und des automatischen Abbruchs der Insulinabgabe zur Vermeidung von Hypoglykämien scheinen diese Systeme hervorragend zur Behandlung des pankreopriven Diabetes geeignet.

## VI) Diabetes bei zystischer Fibrose (CFRD)

Diabetes mellitus bei zystischer Fibrose (engl. „cystic fibrosis related diabetes“ [CFRD]; auch Mukoviszidose) stellt die häufigste extrapulmonale Komplikation bei Patient:innen mit zystischer Fibrose dar [91]. Die Prävalenz steigt mit zunehmendem Alter und liegt bei Erwachsenen bei ca. 50 % [91], bei Patient:innen nach Organtransplantation (Lungen- oder Lebertransplantation) vermutlich noch höher.

Pathophysiologisch steht vor allem die verminderte Insulinsekretion durch die krankheitsspezifische Pankreasfibrose im Vordergrund. Einige Autor:innen beschreiben zudem eine variable Insulinresistenz durch rezidivierende Infekte und wiederholte Glukokortikoidtherapie im Rahmen von Infektexazerbationen [91, 92]. Der CFRD ist assoziiert mit einer Verschlechterung der Lungenfunktion, des Ernährungsstatus sowie letztendlich des Überlebens [93].

Als Screeningmethode wird die Durchführung eines oralen Glukosetoleranztests jährlich ab dem 10. Lebensjahr unter stabilen klinischen Bedingungen empfohlen [91, 94]. Zusätzlich wird ein Screening in bestimmten klinischen Situationen empfohlen: während eines stationären Aufenthalts aufgrund einer Infektexazerbation (Erstellung eines Glukoseprofils mit Nüchtern- und postprandialen Werten), bei enteraler Ernährung sowie bei Frauen mit Kinderwunsch vor der geplanten Schwangerschaft, zwischen der 11. und 13. sowie zwischen der 24. und 28. Schwangerschaftswoche und 6 bis 12 Wochen nach der Entbindung.

Für die Diagnose eines CFRD gelten die allgemeinen Blutzuckergrenzwerte zur Diabetesdiagnose. Zudem wurde wiederholt ein erhöhter 1‑h-postprandialer Wert > 200 mg/dl mit einer klinischen Verschlechterung in Zusammenhang gebracht, weswegen bei Patient:innen mit dieser Form der Glukoseintoleranz („indeterminate glucose tolerance“ [INDET]) in Situationen klinischer Verschlechterung eine vorübergehende Insulintherapie in Erwägung gezogen werden sollte.

HbA_1C_ mit einem Cut-off von 5,5 %, das kontinuierliche Glukosemonitoringsystem (CGM) oder der HOMA-B-Index werden aktuell in Studien evaluiert, können derzeit als Screeningmethoden jedoch noch nicht empfohlen werden [27]. Der Einsatz eines CGM-Systems wurde in einigen Studien empfohlen, ist aber als generelle Screeningmethode noch nicht etabliert. In Zentren mit Erfahrung in Anwendung und Interpretation von CGM-Auswertungen kann CGM ein hilfreiches Mittel zur Beurteilung des Therapieverlaufes und Therapieerfolges darstellen [95].

Die Therapie der Wahl ist jegliche Insulintherapie, wobei sämtliche Therapieformen infrage kommen: Der Insulinbedarf ist meist etwas niedriger als bei Menschen mit Typ-1-Diabetes und wird mit etwa 0,4 IE/kg und Tag bei Jugendlichen, 0,5 IE/kg und Tag bei Erwachsenen und 0,6 IE/kg und Tag bei Patient:innen nach Organtransplantation angegeben [96, 97]. Studien mit oralen Antidiabetika, insbesondere Sulfonylharnstoffen, konnten nur einen vorübergehenden Effekt zeigen und werden derzeit nicht empfohlen. Positive Einzelfallberichte liegen auch zum Einsatz von GLP-1-RA bei gleichzeitig bestehender Adipositas vor [98]. Ziel der Insulintherapie ist vor allem das Erreichen eines anabolen Stoffwechsels, um die Versorgung mit Makronährstoffen und Aufrechterhaltung des Körpergewichts zu sichern [27]. Hinsichtlich diätetischer Empfehlungen unterscheidet sich der CFRD wesentlich von anderen Diabetesformen, da sowohl von einer Kalorien‑, Kohlenhydrat-, und Protein-reduzierten Diät abgeraten und eine protein- und salzreiche Ernährung sogar empfohlen wird [94]. Lediglich „Softdrinks“ sollen in der Ernährung weggelassen werden, da sie zu teils schwer beherrschbaren Blutzuckeranstiegen führen [99].

Die Verlaufskontrolle unterscheidet sich wesentlich von anderen Diabetesformen. Als primärer Parameter gilt der Gewichtsverlauf. Jeder ungewollte Gewichtsverlust bei Patient:innen mit CFRD soll Anlass für eine Therapiekontrolle geben. Ergänzend kann das HbA_1C_ herangezogen werden.

Mit den Erfolgen der Modulatorentherapie zeigt sich jedoch bereits laut Europäischer Registerdaten (ECFSPR Annual Report 2022) ein Rückgang der Untergewichtsrate, und bestehende Ernährungs- und Therapiekonzepte werden in Zukunft bei Zunahme von Übergewicht überdacht werden [100]. Der Einfluss einer Modulatorentherapie auf Entstehung und Verlauf des CFRD dürfte individuell sehr unterschiedlich sein, sodass hierzu aktuell noch keine Information oder Empfehlung gegeben werden kann.

Das therapeutische Ziel unter der primären Insulintherapie liegt bei einem HbA_1c_ < 7 % (53 mmol/mol) bzw. einer „time in range“ (TiR) > 70 % und sollte individuell angepasst werden [96, 101].

Neben den bereits beschriebenen Komplikationen des CFRD (Gewichtsverlust, Verschlechterung des Ernährungszustandes und der Lungenfunktion) spielen von den klassischen diabetischen Spätkomplikationen vor allem mikroangio- und neuropathische Komplikationen eine Rolle, die aufgrund der in den letzten Jahrzehnten deutlich gesteigerten Lebenserwartung bei CF zunehmen werden. Deshalb wird ein jährliches Screening beginnend 5 Jahre nach Diabetesmanifestation empfohlen. Akutkomplikationen wie Hypoglykämien und diabetische Ketoazidose werden beim CFRD relativ selten beobachtet, Letztere kommt aufgrund der residuellen Insulinproduktion nur selten vor.

## VII) Weitere Diabetesformen

Diese seltenen Erkrankungen umfassen infektiöse Formen (kongenitale Röteln) oder auch das Stiffman-Syndrom (auch Stiff-Person-Syndrom genannt). Beim Letzteren handelt es sich um eine Autoimmunerkrankung mit neurologischer Symptomatik, die spontan oder paraneoplastisch auftreten kann.

## VIII) Exokrine Pankreasinsuffizienz (EPI)

Die EPI ist definiert als Abnahme der exokrinen Pankreasfunktion und/oder intraluminaler Aktivität der Pankreasenzyme, sodass die normale Nahrungsmittelverdauung gestört ist. EPI ist mit Malabsorption von Nährstoffen assoziiert und kann zu intestinalen Beschwerden und/oder Nährstoffdefiziten führen [102].

### Prävalenz und Pathogenese

In der Literatur werden EPI-Prävalenzen zwischen 10 und 56 % bei Menschen mit Typ-1-Diabetes angegeben [103–107]. Eine schwere EPI, die durch eine Elastase-1-Konzentration im Stuhl < 100 µg/g definiert ist, wurde dabei bei 10–30 % der Patient:innen festgestellt. Eine EPI ist bei jedem dritten Menschen mit Typ-2-Diabetes beschrieben, wobei mehr als die Hälfte eine schwere Form aufweist [103, 104, 107–109]. Ursächlich findet sich bei T1D eine verminderte Dichte an parasympathischen Axonen im exokrinen Pankreas [110]. Zudem kommt es im Rahmen der Entzündung auch zu einer Störung der Betazellregeneration, was aufgrund des gemeinsamen Ursprungs von exokrinen und endokrinen pankreatischen Vorläuferzellen ebenso zur exokrinen Insuffizienz beiträgt [111–113]. Die Pathophysiologie von EPI bei Typ-1- und Typ-2-Diabetes ist nicht vollständig geklärt, inkludiert aber Inflammation, Fibrose und Steatose des exokrinen Pankreas und Störungen in der Insel-Azinar-Achse [114].

In einer Studie bei Menschen mit Typ-1- und Typ-2-Diabetes wurde eine EPI, definiert als eine fäkale Elastase-1-Konzentration < 200 µg/g, bei 13 % nachgewiesen [115]. In dieser Studie war EPI häufiger bei Typ-1- vs. Typ-2-DM und die Diabetesdauer ein Risikofaktor für EPI. In anderen Studien wurde keine Assoziation zwischen Diabetesdauer und EPI gefunden [105]. In einer Metaanalyse von 17 Studien mit 3662 Menschen mit DM war eine EPI, definiert als fäkale Elastase 1 < 200 µg/g, ebenfalls häufiger bei Typ-1- (38,62 %) als Typ-2-DM (28,12 %) [116].

Die chronische Pankreatitis ist die häufigste Ursache der EPI bei Erwachsenen [117, 118]. EPI ist ein unabhängiger Risikofaktor für Mortalität bei chronischer Pankreatitis [119]. Etwa 85 % der Neugeborenen mit zystischer Fibrose (CF) sind pankreatisch insuffizient (PI) [120]. Neben Pankreasresektionen [121] können auch Magen- oder Dünndarmresektionen durch den Verlust der Sekretin- und Cholecystokinin-Synthese bzw. rascher Magenentleerung mit EPI assoziiert sein, woran auch bei Patient:innen nach bariatrischer Chirurgie gedacht werden sollte [122]. Eine altersbedingte Pankreasatrophie (5 % ab > 70 Jahren, 10 % > 80 Jahren) kann ebenfalls mit EPI einhergehen [123]. Eine akute (vor allem nekrotisierende) Pankreatitis kann ebenfalls mit EPI einhergehen. Bei Pankreaskarzinomen kann durch die Obstruktion des D. pancreaticus ebenso eine EPI entstehen [102].

Andere seltene Ursachen einer EPI sind die hereditäre Hämochromatose, Gastrinome (Inaktivierung von Pankreasenzymen durch Magensäure) und die Zöliakie.

### Klinische Manifestationen bei EPI

Patient:innen mit milder EPI können asymptomatisch sein oder über leichtes abdominelles Unbehagen und Blähungen mit normalem Stuhlgang berichten. Bei schwerer EPI kann es infolge von Fett- und Proteinmaldigestion zu Gewichtsverlust kommen. Eine offenkundige Steatorrhö tritt erst bei Verlust von ca. 90 % der glandulären Funktion auf und geht mit übelriechenden, fettigen Stühlen mit reduzierter Konsistenz, die sich schwer wegspülen lassen, einher. Weitere Symptome sind Blähungen, abdominelle Krämpfe und Flatulenzen. Obwohl klinisch symptomatische Vitaminmangelzustände mit metabolischer Knochenerkrankung oder gestörter Nachtsicht selten sind, sollte ein Mangel an fettlöslichen Vitaminen (ADEK) ausgeschlossen werden [118, 124–126]. Seltener liegt ein Vitamin‑B_12_-Mangel vor (reduzierter intestinaler pH). Als Komplikation der EPI kann eine Sarkopenie bzw. Osteopenie auftreten. Weiters ist das Risiko einer SIBO („small intestinal bacterial overgrowth“) bei EPI erhöht [127]. Eine Assoziation der EPI mit Nierensteinen ist ebenfalls beschrieben [128].

### Screening und Diagnostik der exokrinen Pankreasinsuffizienz

Die Diagnose der EPI basiert auf einer Kombination von Anamnese, Erhebung des Ernährungsstatus und der Pankreasfunktion im entsprechenden klinischen Kontext [102]. EPI sollte bei Patient:innen mit chronischer Diarrhö/Steatorrhö und chronischen Abdominalschmerzen, aber auch bei Patient:innen mit milderen Symptomen wie Blähungen und typischer Bildgebung für chronische Pankreatitis (z. B. Kalzifikationen und/oder Pankreasgangdilatationen und/oder Pseudozysten im Ultraschall, in CT oder MRT) oder Pankreasatrophie suspiziert werden. Einschränkend muss erwähnt werden, dass Symptome weder sensitiv noch spezifisch für EPI sind [102].

Bei entsprechender Symptomatik folgt zur weiteren Diagnostik meist ein indirekter (einfach, günstig) oder direkter Pankreasfunktionstest (Stimulation des Pankreas durch hormonelle Sekretagoga mit nachfolgender Entnahme und Analyse von Duodenalflüssigkeit, z. B. Sekretin-Test: aufwendig, invasiv, schlechte Patient:innentoleranz). Die Bestimmung der Elastase-1-Konzentration im Stuhl mittels Immunassay (indirekter Test) gilt als Standardtest mit einer Sensitivität von etwa 65 % bei milder und bis zu 100 % bei schwerer Form [129]. Eine fäkale Elastase-1 < 200 µg/g gilt als pathologisch, eine fäkale Elastase-1 < 100 µg/g gilt als schwere EPI. Für die Bestimmung der Elastase im Stuhl sollten die geformtesten Stühle eingesendet werden, da falsch niedrige Elastasewerte bei flüssigen Stühlen möglich sind [102]. Die Bestimmung der fäkalen Elastase in mehreren Stuhlproben kann die diagnostische Ausbeute verbessern [128]. Aufgrund der relativ hohen Prävalenz der EPI ist bei gastrointestinaler Beschwerdesymptomatik ein Screening bei Patient:innen mit Diabetes sinnvoll [130–133]. Eine Assoziation zwischen HbA_1c_-Werten mit erniedrigten Elastasewerten ist bei Menschen mit neu diagnostiziertem Typ-2-DM beschrieben [134].

Die differenzialdiagnostisch in Erwägung zu ziehenden Erkrankungen ergeben sich aus der genannten Symptomatik der EPI und umfassen insbesondere die zahlreichen anderen Ursachen einer chronischen Diarrhö. Speziell sind eine autonome Neuropathie des Magen-Darm-Traktes sowie gastrointestinale Nebenwirkungen von Antidiabetika (Metformin, Inkretinanaloga) zu erwägen. Auch Zuckeraustauschstoffe, wie die häufig verwendete Fruktose oder Sorbit, können bei Unverträglichkeit vergleichbare Symptome verursachen. Differenzialdiagnostisch kommt auch eine häufig bei Menschen mit Diabetes vorliegende bakterielle Fehlbesiedlung im Dünndarm (SIBO) in Betracht [135].

Speziell bei Patient:innen mit Typ-1-Diabetes sollte an eine Zöliakie gedacht und diese mittels serologischer Diagnostik (Antikörper gegen Gewebstransglutaminase [tTG]) ausgeschlossen werden. Ein normales Stuhl-Calprotectin ist bei der Unterscheidung zwischen organischen/entzündlichen (z. B. Morbus Crohn, Colitis ulcerosa) und funktionellen gastrointestinalen Erkrankungen (z. B. Reizdarm, funktionelle Dyspepsie) hilfreich. Zusätzlich ist der Ausschluss eines Pankreaskarzinoms (mittels CT/MRT und/oder Endosonographie) wichtig, welches bei Diabetes im Vergleich zur nichtdiabetischen Bevölkerung häufiger auftritt [84]. Die Inzidenz für Pankreaskarzinome ist im 1. Jahr nach Diagnosestellung eines Diabetes mellitus deutlich erhöht mit einem relativen Risiko von 5,38, weshalb gerade zu Beginn einer Diabetesdiagnose besondere Aufmerksamkeit darauf gelegt werden sollte [136].

### Therapie

Die Therapie der EPI besteht in einer ausreichenden Substitution von Pankreasenzymen und sollte allen Patient:innen mit EPI angeboten werden. Die initiale Dosierung der Pankreasenzyme kann abhängig vom Patient:innenalter (Kind vs. erwachsen), Schwere der EPI und Fettgehalt der Nahrung variieren. Die Verabreichung von 40.000 bis 50.000 Lipaseeinheiten mit den Hauptmahlzeiten und die Hälfte dieser Dosis (20.000 bis 25.000 Einheiten) zu Zwischenmahlzeiten entspricht einer üblichen Dosierung bei Erwachsenen. Eine Mitbetreuung durch Diätologinnen ist empfehlenswert. Bei Patient:innen mit EPI unter adäquater Enzymsubstitutionstherapie ist eine Reduktion des Fettgehalts in der Nahrung in der Regel nicht erforderlich. Die in Österreich verfügbaren Pankreasenzympräparate werden aus Schweinepankreas hergestellt. Obwohl Präparate auf pflanzlicher oder Pilzbasis in Entwicklung sind bzw. in manchen Ländern bereits verfügbar sind, ist deren Wirksamkeit nicht gut etabliert [102]. Fettlösliche Vitamine sollten zusammen mit Pankreasenzymen eingenommen werden. Von Nikotinkonsum sollte unbedingt abgeraten werden, da Rauchen ein unabhängiger Risikofaktor für EPI sogar bei Patient:innen ohne vorhergehende Pankreaserkrankung zu sein scheint [137].

## Interessenkonflikt

S. Kaser hat Vortragshonorare bzw. Beraterhonorare erhalten von Novo Nordisk, Eli Lilly, Sanofi, Boehringer Ingelheim, Amarin. S.E. Hofer hat Vortragshonorare bzw. Beraterhonorare erhalten von E. Lilly, Sanofi, Ypsomed. L. Kazemi-Shirazi hat folgende Unterstützungen/Honorare bekommen: Past/present Principal investigator bei Studien gesponsort durch: SigmaTau, Sanofi, Tigenix und Falk; Vortragshonorare erhalten von: MSD, AbbVie, Ferring, Merck Serono/Dr. Falk, Chiesi, Novartis, Roche, Abbott, Phadia Austria/Thermo Fisher Scientific, CSL-Behring, Janssen-Cilag Pharma, Labors.at GmbH, Vertex, ENML Laborbetriebs GmbH, Johnson & Johnson. Kongressunterstützungen von: Mylan, Abbott, MSD, Gilead, Merck Serono/Dr Falk, Novartis, Pfizer, Janssen-Cilag Pharma, Chiesi, Shire, AbbVie, Takeda, Vertex, Astropharma, Konsulent/Berater-Tätigkeit: MSD, Takeda. T. Stulnig hat von folgenden Unternehmen Reiseunterstützung, Honorare für Vorträge, Beratertätigkeit oder Forschungsunterstützungen erhalten: 89bio, Akcea, Amarin, Amgen, AstraZeneca, Boehringer Ingelheim, Eli Lilly, Novo Nordisk, Sanofi. Y. Winhofer-Stöckl hat Vortrags- und Beratungshonorare erhalten von Novo Nordisk, Eli Lilly, Sanofi, Abbott und Dexcom. M.l. Resl hat Vortragshonorare bzw. Beraterhonorare von Eli Lilly, Bayer, Abbott, Dexcom und Boehringer Ingelheim erhalten. C. Ress hat keine potenziellen Interessenkonflikte. H. Sourij gibt folgende Offenlegungen an: Advisory Boards von Amgen, Amarin, Boehringer Ingelheim, Bayer, Eli Lilly, Daiichi Sankyo, Novartis, Novo Nordisk, Sanofi; Honorare für Vortragstätigkeit: Amgen, Amarin, Boehringer Ingelheim, Bayer, Eli Lilly, Daiichi Sankyo, Novartis, Novo Nordisk, Sanofi. F. Aberer hat von folgenden Unternehmen Reiseunterstützung, Honorare für Vorträge, Beratertätigkeit oder Forschungsunterstützungen erhalten: Amgen, Bayer, Sanofi, Novo Nordisk, AstraZeneca und Boehringer Ingelheim. G. Treiber hat Vortragshonorare erhalten von Medtronic, Novo Nordisk, Sanofi. A. Szodl hat von folgenden Unternehmen Reiseunterstützung, Honorare für Vorträge, Beratertätigkeit oder Forschungsunterstützungen erhalten: Sanofi, Novo Nordisk, AstraZeneca, Boehringer Ingelheim und Ascendis Pharma. B. Radlinger hat Vortragshonorare von Amgen und Daiichi Sankyo sowie Kongressunterstützung von Daiichi Sankyo und Novo Nordisk erhalten. E. Binder und A. Luger geben an, dass kein Interessenkonflikt besteht.
